# The Gut Microbiome and Metabolomics Profiles of Restricting and Binge-Purging Type Anorexia Nervosa

**DOI:** 10.3390/nu13020507

**Published:** 2021-02-04

**Authors:** Alessio Maria Monteleone, Jacopo Troisi, Gloria Serena, Alessio Fasano, Riccardo Dalle Grave, Giammarco Cascino, Francesca Marciello, Simona Calugi, Giovanni Scala, Giulio Corrivetti, Palmiero Monteleone

**Affiliations:** 1 Department of Psychiatry, University of Campania “Luigi Vanvitelli”, 80138 Naples, Italy; alessio.monteleone@fastwebnet.it; 2 Theoreo srl, Via Degli Ulivi 3, 84090 Montecorvino Pugliano, Italy; jtroisi@unisa.it (J.T.); scala@hosmotic.com (G.S.); 3 European Biomedical Research Institute of Salerno (EBRIS), Via S. De Renzi, 3, 84125 Salerno, Italy; afasano@mgh.harvard.edu (A.F.); corrivetti@gmail.com (G.C.); 4 Mucosal Immunology and Biology Research Center, Massachusetts General Hospital—Harvard Medical School, Boston, MA 02114, USA; gserena@mgh.harvard.edu; 5 Department of Eating and Weight Disorders, Villa Garda Hospital, Garda, 37016 Verona, Italy; rdalleg@gmail.com (R.D.G.); si.calugi@gmail.com (S.C.); 6 Department of Medicine, Surgery and Dentistry, “Scuola Medica Salernitana”, Neuroscience Section, University of Salerno, 84081 Baronissi, Italy; gcascino@unisa.it (G.C.); francescamarciello81@gmail.com (F.M.)

**Keywords:** restricting anorexia nervosa, binge-purging anorexia nervosa, eating disorders, gut microbiome, fecal metabolomics

## Abstract

Alterations in the gut microbiome and fecal metabolites have been detected in anorexia nervosa (AN), but differences in those profiles between restricting AN (ANR) and binge-purging AN (ANBP) type have not been explored. We made a secondary analysis of our previous data concerning microbiome and metabolomics profiles of 17 ANR women, six ANBP women and 20 healthy controls (HC). Twelve fecal metabolites differentiating ANR patients, ANBP patients and HC were identified. Both patient groups showed decreased intra-individual bacterial richness with respect to healthy controls (HC). Compared to ANR subjects, ANBP patients had a significant increase in relative abundances of *Bifidobacterium*, Bifidobacteriaceae, Bifidobacteriales, and Eubacteriacae and a significant decrease in relative abundances of *Odoribacter*, *Haemophilus*, Pasteurellaceae, and Pasteurellales. The heatmaps of the relationships of selected fecal metabolites with microbial families showed different structures among the three groups, with the heatmap of ANBP patients being drastically different from that of HC, while that of ANR patients resulted more similar to HC. These findings, although preliminary because of the relatively small sample size, confirm the occurrence of different gut dysbiosis in ANR and ANBP and demonstrate different connections between gut microorganisms and fecal metabolites in the two AN types.

## 1. Introduction

It is widely acknowledged that in both animals and humans, the gut microbiota plays a pivotal role in several physiological processes, which may have a potential influence on human behavior and are essential for the maintenance of body homeostasis [1,2,3]. Indeed, increasing evidence indicates that alterations in the composition of gut microbiota are associated with various human diseases [4,5], and bidirectional communication between the gastrointestinal tract and the brain has been documented with a pivotal modulatory role played by the gut microbiota [6]. Several environmental factors affect the composition of gut microbiota and, among them, the type and the composition of the individual’s diet seem to be major determinants. Indeed, variations in both short- and long-term dietary patterns have been shown to rapidly affect the composition of the gut microbiota [7,8]. Since intestinal microbiota produce an array of bioactive metabolites capable of entering systemic circulation and exerting profound effects on various physiological processes [1,2,3], changes in its composition may affect host physiology. In particular, alterations in stress response, brain neurochemistry, and behavior are indicative of a reduction in anxiety have been detected in mice lacking intestinal microbial load [3], and a role for the gut microbiota in the regulation of host appetite and body weight has been widely demonstrated [9,10,11].

Anorexia nervosa (AN) is a psychiatric disorder characterized by abnormal eating behaviors, such as fasting, food restriction, and/or binge-eating with compensatory behaviors (e.g., self-induced vomiting, and diuretic and laxative misuse, and excessive exercising) leading to bodyweight loss and physical complications with potentially life-threatening consequences [12]. Although the exact pathophysiological mechanisms of AN are not known yet, an implication of gut microbiota has been hypothesized. Indeed, in the last decade, different research groups have documented gut microbiota dysbiosis in acutely ill and/or partially weight-restored AN patients, although findings were not consistent among the studies [13,14]. Even if those reported changes in microbiota composition of AN patients may be secondary to their dietary restriction, starvation, and/or binge-eating with associated compensatory behaviors, it is likely that they may lead to the production of bacterial metabolites, which may promote the maintenance of AN aberrant behaviors and symptoms. Therefore, the assessment of the associations of fecal metabolites with gut microbiota profiles of patients with AN may provide a more comprehensive insight into the role of intestinal microbiota in the pathophysiology of AN. In this line, we have recently demonstrated that, in acutely ill AN women, gut microorganisms were associated with fecal metabolites differently from healthy women and that most of those associations had divergent directions in the acute phase with respect to the weight-restored phase of the illness [15,16]. However, two different types of AN have been identified: (i) the restricting type (ANR) in which weight loss is accomplished primarily through dieting, fasting, and/or excessive exercise and (ii) the binge-eating/purging type (ANBP) in which the individuals engage in recurrent episodes of binge eating or purging behaviors such as self-induced vomiting or misuse of laxatives, diuretics, or enemas [17]. Therefore, it is likely that the different eating and purging behaviors of ANR and ANBP types differently affect gut microbiota and fecal metabolomic profiles of AN people. In this regard, Mack et al. [18] reported a significantly different gut community structure of ANR versus ANBP type, but they did not provide a comprehensive description of such a difference. Moreover, to the best of our knowledge, so far, no study has investigated the fecal metabolomic profile and its association to gut microbiota in ANR versus ANBP individuals. Therefore, in the present study, we made a secondary analysis of our previous data [15,16] to characterize fecal metabolites, gut microorganisms, and their interactions in ANR and ANBP patients. On the basis of literature data, we hypothesized that fecal metabolomic and microbiome profiles of the two AN types should be substantially different.

## 2. Materials and Methods

This study is a secondary analysis of data from our previous studies [15,16] investigating the microbiome and metabolomic profiles and their interactions in women meeting the Diagnostic and Statistical Manual of Mental Disorders (DSM-5) criteria for AN, as assessed by the Structured Clinical Interview for DSM-5, Clinician Version (SCID-5-CV), who were consecutively admitted to the Department of Eating and Weight Disorders of Villa Garda Hospital (Garda—VR, Italy), and in healthy women. Exclusion criteria for all the participants were: (1) presence of diarrhea in the last month; (2) lifetime history of celiac disease, surgery on the gastrointestinal tract, inflammatory bowel disease, bowel tumors, pathologies leading to malabsorption, chemo or radiotherapy treatments (even if due to pathologies other than the colon); (3) chronic diseases and/or endocrine/metabolic disorders not related to AN; (4) treatment with antibiotics in the last three months; (5) intake of probiotics, enemas, laxatives or other drugs, including psychotropic medications, in the last two months. None of the participants was using oral contraception or was receiving hormonal therapies; none of them were smokers. Data from six ANBP women, 17 ANR women, and 20 healthy women were available for this secondary analysis.

Participants underwent a clinical assessment including the measurement of eating disorders and general psychopathology using the validated Italian version of the Eating Disorder Examination (EDE) interview [19,20] and the validated Italian version of the Brief Symptom Inventory (BSI), which is a short version of the Symptom Checklist-90 [21,22]. The SCID-5-CV was used to assess current psychiatric comorbidity [23]. Patients collected a fecal sample within one week from their admission into the inpatient unit and before starting any treatment program when they were receiving a standardized diet. Healthy controls (HC) collected fecal samples after one week of a standardized diet. Fresh stool samples were aliquoted in two vials (one vial with RNA later solution and one without solution) and immediately frozen at −80 °C until assayed.

In the week preceding the collection of the fecal sample, AN patients received a standardized diet of 1500 kcal/day with 54.44% carbohydrate, 17.00% protein, and 28.56% lipid, while healthy women received a standardized diet of 2000 kcal/day with 45% carbohydrate, 18% protein, and 35% lipid.

Each participant signed a written informed consent form. The study was carried out in accordance with the ethical principles of the declaration of Helsinki and approved by the University of Salerno’s ethics committee (reference number: 31 r.p.s.o., 12 April 2017).

### 2.1. Microbiome Analysis

Microbiome sequencing was performed at the Massachusetts General Hospital NGS Core Facility in Boston (MA, USA). Detailed methods were reported in Monteleone et al. [15]. Briefly, after DNA extraction, V4 regions of the 16S rRNA gene were amplified using a dual barcode system using a fusion primer 515F and 806R. Electrophoretic confirmed amplified were purifies using Quiaquick PCR purification kit (Qiagen, Germantown, MD, USA). After amplicon concentration measurement using Quant-IT Picogreen dsDNA Assay Kit (Thermo Fisher, Waltham, MA, USA), they were sequenced on the Illumina MiSeq v2 using 500 cycles.

### 2.2. Metabolomic Analysis

Stool metabolomic profiling analysis was performed according to Monteleone et al. [15,16]. Briefly, fecal metabolome was extracted and purified using a MetaboPrep GC kit (Theoreo srl—Montecorvino Pugliano, SA, Italy). Profiles were acquired using Gas Chromatograph Mass Spectrometry GCMS-2010 (Shimadzu, Kyoto, Japan). A total of 224 endogenous metabolites involved in energy metabolism, lipid metabolism, and amino acid metabolism were detected sequentially, according to an untargeted metabolomic approach.

### 2.3. Statistical Analysis

Demographic and clinical data were analyzed by using R software [24] (version 4.0.3) using R-study IDE (version 1.3.959). The data distribution was tested using the Shapiro–Wilks test. Since data resulted normally distributed, intergroup differences were tested by one-way ANOVA followed by Tukey’s post hoc test. The significance was set at *p* < 0.05.

Microbiome data analysis was performed according to Monteleone et al. [16]. Briefly, reads were preprocessed using the MICCA pipeline (ver. 1.7.2) [25,26]. Sequence clusterization (97% pairwise identity cutoff) allows assigning the operational taxonomic units (OTUs) that were classified using RDP classifier version 2.12 on 16S data [27]. PyNAST (ver. 1.2.2), based on the Greengenes database [28], was used for sequence alignment. Rarefaction was used to reduce the sampling inhomogeneity. Alpha- (within-sample richness) and beta- (between-sample dissimilarity) diversity were estimates using MicrobiomeAnalystR package (ver. 0.0.0.9000) [29]. Linear discriminant effect size (LEfSe) analysis was also performed to find taxa differentially represented among groups.

The supervised machine learning model was based on partial least square discriminant analysis (PLS-DA) according to our previous report [15]. Briefly, analyses were performed using internal standard peak area normalized and chromatogram using MetaboPredict software version 1.2.1 (Theoreo srl). Semi-quantitative data were first aligned, then autoscaled and log-transformed. Models were cross-validated and subjected to a permutation test to verify the lack of overfitting. To manage the class imbalance (six ANBP women vs. 17 ANR women vs. 20 healthy controls) the metacost algorithm [30] was used using the class dimension as a cost matrix. Data dimensionality reduction was performed using genetic algorithm-based selection [31]. Moreover, based on PLS-DA models, variables important in projection (VIP) scores were calculated for each metabolite. Furthermore, one-way ANOVA with Tukey’s post hoc test was used to evaluate metabolite concentration differences among patients and healthy participants. Metabolites with a VIP-score higher than 1.5, or with a *p*-value < 0.05 evaluated using the ANOVA were further confirmed with an independent analytical standard as reported in the Metabolomics Standard Initiative (level metabolics standards initiative (MSI) = 1) [32].

Pathway analysis was conducted using the MetPa algorithm [33]. In the context of pathway analysis, we tested if compounds involved in a particular pathway were enriched compared to random hits using the hypergeometric test. Node centrality (a measure estimation of node importance) was done using the betweenness centrality. It measures the number of shortest paths going through the node. Since the metabolic networks are directed, we used the relative betweenness centrality for a metabolite as an important measure. The betweenness centrality measure focuses on global network topology.

Microbiome and metabolomics data were correlated using heatmaps representation based on Spearman rank correlation coefficients for each pairwise combination of microbial Genus abundances and metabolite semi-quantitative results. Metabolite selection was carried out according to the genetic algorithm as previously reported [15]. Both metabolites and bacteria were clustered.

## 3. Results

### 3.1. Clinical Characteristics

As shown in Table 1, patients with ANR did not significantly differ from those with ANBP in body mass index (BMI) and severity of both eating disorder and general psychopathology. ANR women were younger than ANBP ones. Both patient groups had significantly lower BMI and higher psychopathological measures compared to HC.

### 3.2. Metabolomics

**Figure 1:**
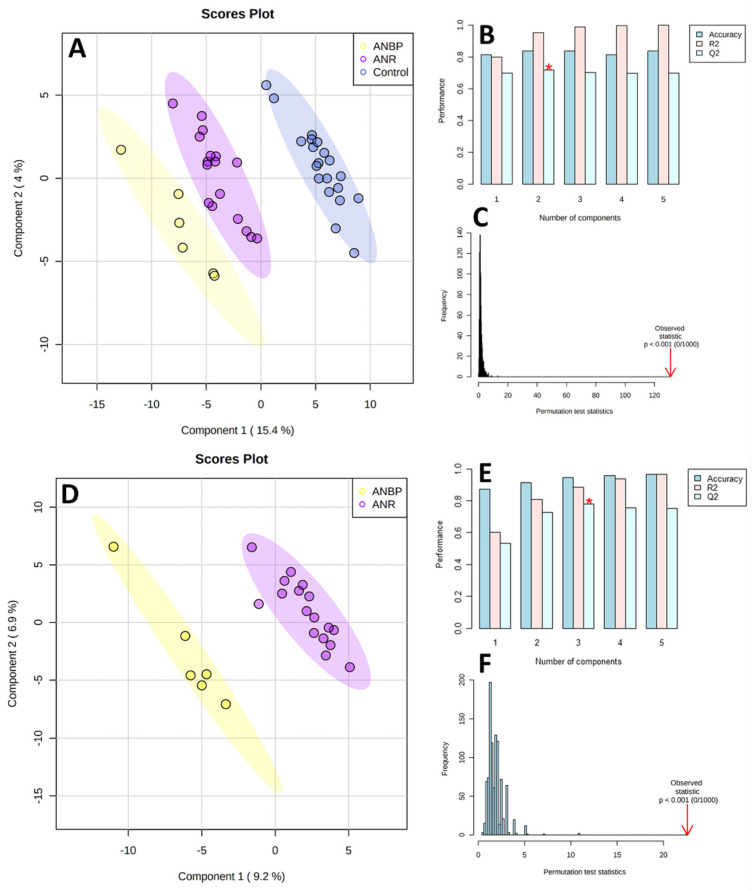
Partial least square discriminant analysis (PLS-DA) models to discriminate healthy controls (CTRL), restricting (ANR), and binge-purging (ANBP) anorexia nervosa patients (**A**) and ANBP and ANR patients (**D**). The explained variance of each component is shown in brackets on the corresponding axis. (**B**,**E**) Accuracy, R^2^, and Q^2^ results estimate via cross-validation. Q^2^ is an evaluation of the predictivity ability of the model. In each cross-validation, the predicted data are compared with the original data, and the sum of squared error is calculated. The better prediction performances are provided by the model, with three components providing for the higher Q^2^ (marked with a red star). (**C**,**F**) Permutation test results based on 1000 iterations.

As shown in Figure 1, the PLS-DA score plots clearly differentiated ANBP and ANR patients and HC with no overlap. The PLS-DA performance, evaluated by means of cross-validation and permutation test, showed no overfitting: Q^2^ = 0.763, R^2^ = 0.961, Accuracy = 0.824, *p* < 0.0001 for the 3-class model, and Q^2^ = 0.798, R^2^ = 0.912, Accuracy = 0.973, *p* < 0.0001 for the 2-class model.

**Figure 2:**
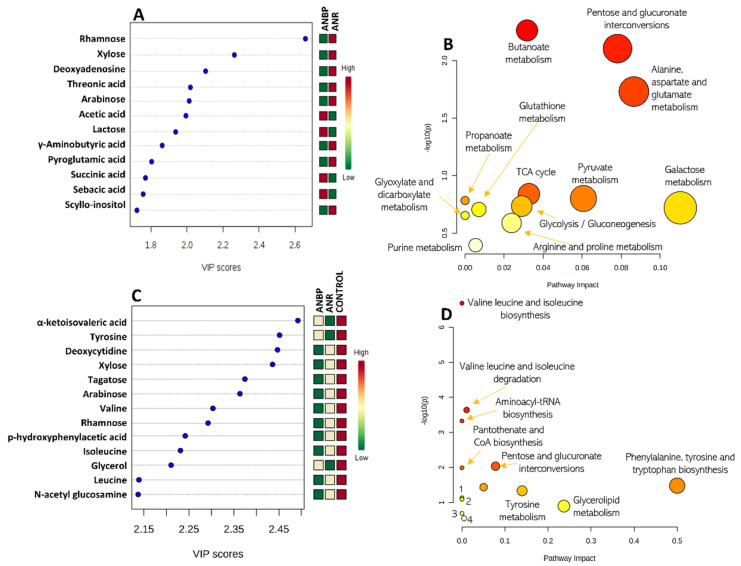
(**A**) Metabolites showing a variables importance in projection (VIP) score higher than 1.5 in a partial least squares discriminant analysis (PLS-DA) model separating restricting (ANR) and binge-purging (ANBP) anorexia nervosa patients. (**C**) Metabolites showing a VIP score higher than 1.5 in the PLS-DA model separating ANBP patients vs. ANR patients vs. healthy controls. Colored boxes on the right indicate the relative amount of the corresponding metabolite in each group under study. (**B**,**D**) Metabolite enriched pathway analysis from the selected metabolites.

The 3-class model separating ANBP and ANR patients and HC identified 13 metabolites with a VIP score > 1.5: α-ketoisovaleric acid, tyrosine, deoxycytidine, xylose, tagatose, arabinose, valine, rhamnose, p-hydroxyphenylacetic acid, isoleucine, glycerol, leucine, and n-acetyl glucosamine (Figure 2A). The 2-class model, separating ANBP and ANR patients identified 12 metabolites with a VIP score > 1.5 (Figure 2A): rhamnose, xylose, deoxyadenosine, threonic acid, arabinose, acetic acid, lactose, γ-aminobutyric acid, pyroglutamic acid, succinic acid, sebacic acid, and scyllo-inositol.

Based on Principal Component Analysis (PCA) on the ANR and ANBP patients (data not shown), the first 22 principal components explained 95% of the total variance. All relevant metabolites with VIP-score higher than 2.0 and/or significantly different among ANR and ANBP patients and healthy controls using ANOVA are reported in Table 2.

The metabolic pathway analyses of the VIP-selected metabolites are summarized in Figure 2B,D. The PLS-DA 3-class model showed that the most relevant involved metabolic pathways were: valine, leucine and isoleucine biosynthesis and degradation, aminoacyl-tRNA biosynthesis, pantothenate biosynthesis, pentose and glucuronate interconversions, phenylalanine, tyrosine and tryptophan biosynthesis, ubiquinone and other terpenoid-quinone biosynthesis, and tyrosine, glycerolipid, phenylalanine, galactose, and pyrimidine metabolism. In the PLS-DA 2-class model, there was a clear-cut interaction of several pathways involving pentose and glucuronate interconversions, citrate cycle, glycolysis/gluconeogenesis, and the metabolism of butanoate, alanine aspartate, glutamate, pyruvate, propanoate metabolism, galactose, glutathione, glyoxylate, dicarboxylate, arginine, proline, and purine.

### 3.3. Microbiome

**Figure 3:**
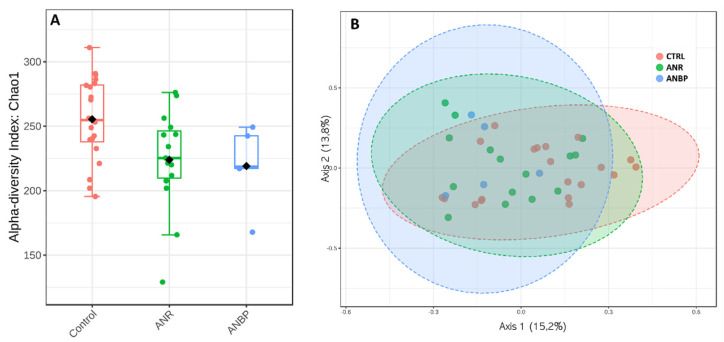
(**A**) Alpha diversity according to Chao index in control (CTRL), restricting (ANR), and binge-purging (ANBP) anorexia nervosa patients. (**B**) Microbiome beta-diversity represented in terms of non-metric multidimensional scaling.

With respect to HC, alpha-diversity measured by Chao index (Figure 3A) showed a significantly reduced value in ANR patients (*p* = 0.02) and a trend toward a significant reduction in ANBP ones (*p* = 0.07) while no significant difference emerged between ANR and ANBP groups (*p* = 0.88). Beta diversity, reported in terms of non-metric multidimensional scaling, showed no significant difference among the analyzed samples resulting in a *p* = 0.72 based on Permanova evaluation (Figure 3B).

Analysis of bacteria amounts at phylum levels showed that Actinobacteria were significantly less abundant in ANR compared to ANBP patients (*p* = 0.009), and Verrucomicrobia resulted higher in ANR women than in healthy controls (*p* = 0.02). No significant difference in terms of Bacteroidetes to Firmicutes ratio was detected among the groups.

**Figure 4:**
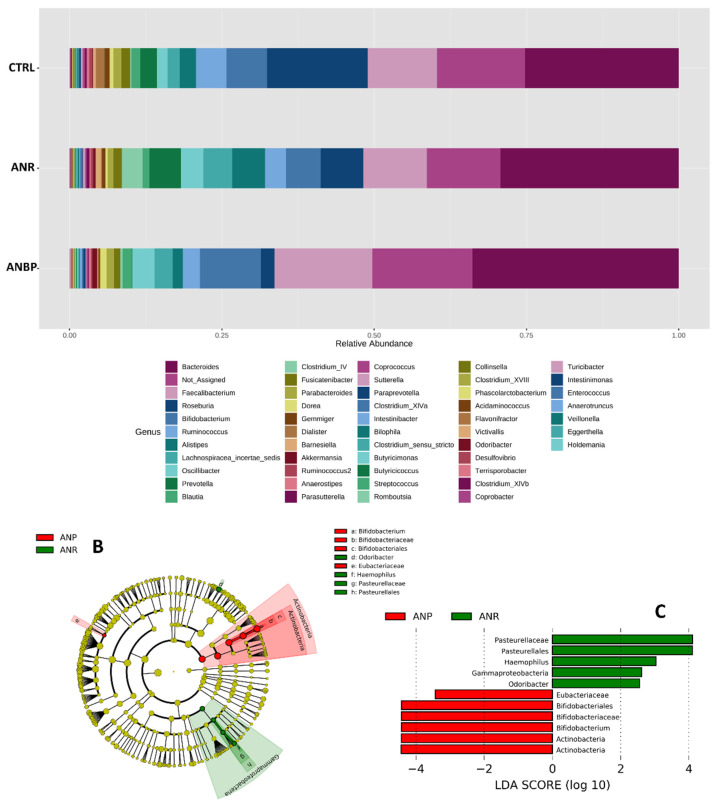
(**A**) Bacteria abundances in terms of genus in healthy subjects (CTRL), restricting (ANR) and binge-purging (ANP) anorexia nervosa patients during the acutely ill phase. (**B**) Cladograms generated by linear discriminant effect size (LEfSe) indicating differences in the bacterial taxa between ANR and ANP patients. Nodes in red indicate taxa that were enriched in ANBP, while nodes in green indicate the opposite trend. (**C**) Linear Discriminant Analysis (LDA) scores for the bacterial taxa differentially abundant between the two groups. Only taxa having a *p* < 0.01 were shown.

Bacteria amounts at the genus level in the three study groups are shown in Figure 4A. LEfSe analysis revealed that ANBP patients had a significant increase in relative abundances of *Bifidobacterium*, Bifidobacteriaceae, Bifidobacteriales, and Eubacteriacae as well as a significant decrease in relative abundances of *Odoribacter*, *Haemophilus*, Pasteurellaceae, and Pasteurellales (Figure 4B,C).

### 3.4. Microbiome-Metabolomics Integration

**Figure 5:**
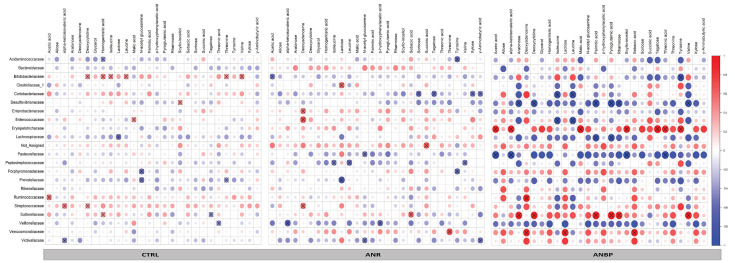
Heatmaps reporting the Spearman correlation coefficients (r) for each pairwise combination of microbial Family abundances and metabolite levels evaluated in patients with restricting anorexia nervosa (ANR), patients with binge-purging anorexia nervosa (ANBP), and healthy controls (CTRL). Crosses show the correlation coefficients with *p* < 0.05.

Correlation coefficient analysis of the relationships between metabolites selected by VIP-score and ANOVA on the one side and microbial families on the other side showed different frameworks among the groups (Figure 5). In particular, 43%, 44%, and 47% of the 616 analyzed correlations were positive for healthy subjects, ANBP and ANR patients, respectively. Moreover, in healthy controls, 3.2% of the analyzed correlations were statistically significant (*p* < 0.05); this figure was 3.7% in ANR patients and 5.5% in ANBP patients. Finally, in ANR and ANBP patients, 53% and 49% of the 616 correlations, respectively, showed the same trend (positive or negative) as in healthy controls in terms of rho coefficient, while this figure was 56% in the comparison between ANR and ANBP patients.

## 4. Discussion

In the present study, we made a secondary analysis of data from previously characterized fecal microbiome and metabolomics profiles of women with AN and HC [15,16] to verify whether those profiles differed between patients with acute ANR type and those with acute ANBP type. We found that a PLS-DA 3-class model identified 13 fecal metabolites with a VIP score higher than 1.5 that were able to differentiate between patients with ANR, those with ANBP, and HC with no overlap among the three groups. Moreover, a PLS2-DA 2-class model identified 12 fecal metabolites with a VIP score higher than 1.5 that were able to differentiate between patients with ANR from those with ANBP. Furthermore, by adopting two separate metabolite selection strategies with different statistical approaches (PLS-DA and ANOVA), differences in some fecal metabolites emerged among the three groups. In particular, focusing on fecal metabolites showing a statistically significant difference by ANOVA, some substances (i.e., deoxycytidine, isoleucine, malic acid, n-acetyl-glucosamine, palmitic acid, rhamnose, sorbose, tagatose, and xylose) showed a statistically significant reduction in both patient groups with respect to healthy controls while some others (i.e., allose, glycerol, homogentistic acid, p-hydroxyphenylacetic acid, succinic acid, and threonine) showed statistically significant opposite changes in ANR and ANBP with respects to controls. Finally, compared to ANR patients, ANBP ones showed significantly lower levels of deoxycytidine, isoleucine, malic acid, rhamnose, and xylose but significantly higher levels of n-acetyl-glucosamine, palmitic acid, sorbose, and tagatose. Previous studies that have investigated the metabolomic signature of AN have been focused on targeted metabolites, which are believed possible biomarker of the disease on the basis of specific pathogenetic hypotheses, and have reported significant changes in very long-chain fatty acids [34], steroid metabolome [35], polyunsaturated fatty acids [36,37] and other chemicals [38]. Here we report fecal metabolomics profiles of ANR and ANBP patients as resulting from an untargeted metabolomic approach, which, differently from the targeted methodology, measures as many metabolites as possible. Amid the fecal metabolites that we found statistically different among the groups, the sugars rhamnose, tagatose, sorbose, and xylose resulted uniformly decreased in both patient groups compared to HC with rhamnose and xylose being significantly lower in ANBP than in ANR patients and sorbose and tagatose being significantly higher in ANBP than in ANR patients. These differences may be ascribed to the abnormal feeding of AN patients that relies on energy sources different from those of healthy individuals since they typically avoid the ingestion of sugar-rich food and/or high-fat food [39,40,41]. Moreover, the metabolic pathway analyses of the VIP-selected metabolites identified several metabolic pathways potentially and differently deranged in ANR and ANBP patients. All these findings confirm that ANR and ANBP patients show a profound perturbation in fecal metabolites with respect to healthy women and demonstrate for the first time that the two AN types differ in their fecal metabolomics profiles.

As suggested previously [15], the detected changes in fecal metabolites in patients with AN may result from either their chronic malnutrition and/or changes in their gut microbiota composition. With respect to the first assumption, it has been demonstrated that the diet of women with acute ANR or ANBP differs from that of healthy women in both quantitative and qualitative composition [41]; furthermore, ANBP women not only ingest large amounts of nutrients in their binge-eating episodes but also regurgitate amounts of ingested food [17], which may lead to peculiar nutritional aberrations. These nutritional diversities between the two AN types and HC and between ANR and ANBP individuals may explain part of the observed differences in fecal metabolomics profiles of our ANR and ANBP women. Moreover, present findings also support our second assumption, since profound and distinct changes in the gut microbiome composition and in the relationships between gut bacteria and fecal metabolites were detected between ANR and ANBP women and healthy controls.

In particular, with respect to HC, the intra-individual bacterial richness (alpha-diversity) was reduced in both ANR and ANBP groups, although only in the former reached a statistically significant threshold likely because of the relatively low number of ANBP individuals. The inter-individual difference in gut microbiome composition (beta-diversity) did not statistically differ among the three groups. These data are in line with previous findings from both our group [16] and other research groups [42,43,44] and show for the first time that acute ANR and ANBP patients do not differ in these gut microbiome indices.

At taxonomy levels, the most relevant findings emerged in the comparison of the gut microbiome composition between ANR and ANBP patients. In fact, with respect to ANR patients, ANBP patients exhibited a significant increase in relative abundances of *Bifidobacterium*, Bifidobacteriaceae, Bifidobacteriales, which represent the genus, family, and order levels in the Actinobacteria phylum, and a significant decrease in the relative abundances of *Haemophilus*, Pasteurellaceae and Pastuerellales, which represent the genus, family and order levels in the Proteobacteria phylum. Moreover, ANBP patients had a significantly higher abundance of the genus *Odoribacter* and a lower abundance of the family Eubacteriaceae, which belong to the Bacterioidetes and Firmicutes phyla, respectively. Although these last findings support changes in the relative abundances of those phyla, contrary to our previous results [16], no significant differences emerged in the Bacteroidetes-to-Firmicutes ratio between ANR and ANBP group and between each patient group and healthy controls. This discrepancy was likely due to the relatively small sample sizes of our patient groups resulting from this secondary analysis. Anyway, consistent with our findings, Mack et al. [18] reported a different gut microbiome structure between acute ANR and ANBP patients, but they did not provide a comprehensive analysis of that difference. Therefore, our findings add to the current literature on microbiome composition in AN and provide, for the first time, a detailed microbiome differentiation between the two subtypes of AN.

The heatmaps of the relationships of the 28 selected fecal metabolites with bacteria families showed different structures among the three groups. Indeed, by global inspection, it is evident that the heatmap of ANBP patients was drastically different from that of healthy controls, whereas that of ANR patients was more similar to healthy controls, which suggests a profound perturbation of the gut bacteria and fecal metabolites interactions in AN with some relevant differences between ANR and ANBP patients. Indeed, 44% of the interactions between microbial families and fecal metabolites had an opposite direction in the two AN subtype groups, which supports divergent relationships between gut bacteria and metabolite production (positive correlation) or consummation (negative correlation) in patients with ANR or ANBP. Previous studies have explored concomitantly the composition of the gut microbiome and the fecal concentrations of short-chain fatty acids (SCFA), such as propionate, valerate, butyrate, and/or branched-chain fatty acids (BCFA), such as isovalerate and isobutyrate in AN patients and, although results were not consistent, have suggested possible links between alterations in the gut microbiome and changes in the final products of carbohydrates (SCFA) and protein (BCFA) fermentation by gut bacteria [18,44,45]. Our findings expand such knowledge to the interactions of gut bacteria with several different metabolites and demonstrate for the first time that these interactions not only change in AN but they also differ between the two types of AN.

The main limitation of this secondary analysis study is that subgrouping patients according to the AN subtype resulted in two relatively small patient samples, and this could have affected present results. Thus, these findings should be considered preliminary and need confirmation by future studies with larger patient samples. Notwithstanding, this study is the first one that investigated the differences in the gut microbiome and fecal metabolites between ANR and ANBP patients and present findings, if confirmed, may contribute to further differentiate the two types of AN, adding to the current evidence of clinical and biological diversities of binge-purging and restrictive types of eating disorders [46,47,48].

## 5. Conclusions

We have found distinct perturbations in fecal metabolomics and microbiome profiles of acute ANR patients compared to acute ANBP patients. Moreover, we have provided evidence that the connections between gut bacteria and some fecal metabolites significantly differ between ANR and ANBP women and with respect to HC. These findings, although preliminary because of the relatively small sample size, confirm the occurrence of different gut dysbiosis in ANR and ANBP type and prove for the first time that different connections between gut microorganisms and fecal metabolites can be traced in the two subtypes of AN. Further studies need to establish whether these changes are an epiphenomenon of the altered eating behaviors or if they may have a role in the pathophysiology of AN representing possible therapeutic targets. Indeed, preliminary findings suggest that intervention aiming at correcting the microbiota modifications of AN patients by fecal microbiota transplantation may improve the fecal metabolome and/or the underweight condition [49,50]. Present data support the idea that such therapeutic interventions should also be targeted to the type of AN.

## Figures and Tables

**Table 1:** Clinical characteristics of the study sample. Data are expressed as mean ± SD.

	Women with ANR (*n* = 17)	Women with ANBP (*n* = 6)	Healthy Women (*n* = 20)	F_2,40_	*p*-Value
Age, yrs	20.5 ± 3.1	25.2 ± 5.2	23.0 ± 3.3	4.94	0.02
Body weight, kg	38.6 ± 6.0	39.8 ± 4.3	55.2 ± 6.3	39.59	3.29 × 10^−10^
BMI	15.0 ± 1.8	14.7 ± 1.5	20.3 ± 1.4	59.29	1.08 × 10^−12^
EDE restraint	4.5 ± 0.9	4.4 ± 1.2	0.2 ± 0.6	146.07	4.12 × 10^−19^
EDE eating concern	3.7 ± 1.1	3.8 ± 1.3	0.1 ± 0.4	91.61	1.17 × 10^−15^
EDE weight concern	4.2 ± 1.5	3.7 ± 0.8	0.3 ± 0.7	63.55	3.82 × 10^−13^
EDE shape concern	4.8 ± 1.3	4.7 ± 1.5	0.5 ±1.0	66.19	2.05 × 10^−13^
BSI somatization	1.8 ± 0.9	2.5 ± 1.1	0.1 ± 0.2	36.45	9.69 × 10^−10^
BSI obsessive-compulsive	2.2 ± 0.2	3.0 ± 0.3	0.3 ± 0.4	43.26	9.96 × 10^−11^
BSI interpersonal sensitivity	2.7 ± 0.8	3.3 ± 0.8	0.1 ± 0.2	116.57	2.05 × 10^−17^
BSI depression	2.7 ± 0.7	3.2 ± 0.5	0.2 ± 0.2	145.62	4.34 × 10^−19^
BSI anxiety	2.6 ± 0.8	3.3 ± 0.5	0.4 ± 0.2	93.17	8.83 × 10^−16^
BSI hostility	1.6 ± 0.8	2.4 ± 1.2	0.2 ± 0.3	27.80	2.69 × 10^−8^
BSI phobic anxiety	1.5 ± 1.0	1.9 ± 0.9	0.1 ± 0.1	24.78	9.94 × 10^−8^
BSI paranoid ideation	1.8 ± 0.9	2.3 ± 0.8	0.1 ± 0.3	36.55	9.37 × 10^−10^
BSI psychoticism	2.1 ± 0.8	2.6 ± 0.9	0.1 ± 0.2	64.62	2.95 × 10^−13^

SD: standard deviation; ANR = restricting anorexia nervosa type; ANBP = binge-purging anorexia nervosa type; BMI = Body mass index; EDE = Eating Disorder Examination Interview; BSI = Brief Symptom Inventory.

**Table 2:** Fecal metabolite changes with respect to healthy controls in women with restrictive anorexia nervosa (ANR) and binge-purging anorexia nervosa (ANBP).

Metabolite	HMDB Code	ANR	ANBP	Selection Criteria
Acetic acid	HMDB0000042	↓	↑	PLS-DA-2classes
Allose	HMDB0001151	↓	↑	ANOVA
alpha-ketoisovaleric acid	HMDB0000019	↓	↑	PL-SDA-3classes
Arabinose	HMDB0029942	↓	↓↓	PLS-DA-3classes, PLS-DA-2classes
Deoxyadenosine	HMDB0000101	↓	↓↓	PLS-DA-2classes
Deoxycytidine	HMDB0000014	↓	↓↓	ANOVA, PLS-DA-3classes
Glycerol	HMDB0000131	↓	↑	ANOVA, PLS-DA-3classes
Homogentistic acid	HMDB0000130	↓	↑	ANOVA
Isoleucine	HMDB0000172	↓	↓↓	ANOVA, PLS-DA-3classes
Lactose	HMDB0000186	↓	↑	PLS-DA-2classes
Leucine	HMDB0000687	↓	↓↓	PLS-DA-3classes
Malic acid	HMDB0000156	↓	↓↓	ANOVA
*N*-acetyl glucosamine	HMDB0000803	↓↓	↓	ANOVA, PLS-DA-3classes
Palmitic acid	HMDB0000220	↓↓	↓	ANOVA
p-hydroxyphenylacetic acid	HMDB0000020	↓	↑	ANOVA, PLS-DA-3classes
Pyroglutamic acid	HMDB0000267	↓	↓↓	PLS-DA-2classes
Rhamnose	HMDB0000849	↓	↓↓	ANOVA, PLS-DA-3classes PLSDA-2classes
Scyllo-inositol	HMDB0006088	↓	↑	PLS-DA-2classes
Sebacic acid	HMDB0000792	↓	↑	PLS-DA-2classes
Sorbose	HMDB0001266	↓↓	↓	ANOVA
Succinic acid	HMDB0000254	↓	↑	ANOVA, PLS-DA-2classes
Tagatose	HMDB0003418	↓↓	↓	ANOVA, PLS-DA-3classes
Threonic acid	HMDB0000943	↓	↑	PLS-DA-2classes
Threonine	HMDB0000167	↓	↑	ANOVA
Tyrosine	HMDB0000158	↓	↑	PLS-DA-3classes
Valine	HMDB0000883	↓	↓↓	PLS-DA-3classes
Xylose	HMDB0000098	↓	↓↓	ANOVA, PLS-DA-3classes PLSDA-2classes
γ-Aminobutyric acid	HMDB0000112	↓	↓	PLS-DA-2classes

↑ increased or ↓ decreased compared to healthy controls; ↓↓ decreased compared to both healthy controls and the other AN subtype. Differences were statistically significant for those metabolites selected by analysis of variance (ANOVA). PLS-DA = partial least square discriminant analysis; ANR = restricting type anorexia nervosa; ANBP = binge-purging type anorexia nervosa.

## Data Availability

The data presented in this study are available on request from the corresponding author.
